# Rational Development and Characterization of a Ubiquitin Variant with Selectivity for Ubiquitin C-Terminal Hydrolase L3

**DOI:** 10.3390/biom12010062

**Published:** 2022-01-01

**Authors:** Chad S. Hewitt, Chittaranjan Das, Daniel P. Flaherty

**Affiliations:** 1 Department of Medicinal Chemistry and Molecular Pharmacology, College of Pharmacy, Purdue University, West Lafayette, IN 47907, USA; hewitt8@purdue.edu; 2 Department of Chemistry, College of Science, Purdue University, West Lafayette, IN 47907, USA; cdas@purdue.edu; 3 Purdue Center for Cancer Research, Hanson Life Sciences Research Building, Purdue University, West Lafayette, IN 47907, USA; 4 Purdue Institute for Drug Discovery, Purdue University, West Lafayette, IN 47907, USA

**Keywords:** UCHL3, ubiquitin variants, DUBs, activity-based probes

## Abstract

There is currently a lack of reliable methods and strategies to probe the deubiquitinating enzyme UCHL3. Current small molecules reported for this purpose display reduced potency and selectivity in cellular assays. To bridge this gap and provide an alternative approach to probe UCHL3, our group has carried out the rational design of ubiquitin-variant activity-based probes with selectivity for UCHL3 over the closely related UCHL1 and other DUBs. The approach successfully produced a triple-mutant ubiquitin variant activity-based probe, UbV^Q40V/T66K/V70F^-PRG, that was ultimately 20,000-fold more selective for UCHL3 over UCHL1 when assessed by rate of inactivation assays. This same variant was shown to selectively form covalent adducts with UCHL3 in MDA-MB-231 breast cancer cells and no reactivity toward other DUBs expressed. Overall, this study demonstrates the feasibility of the approach and also provides insight into how this approach may be applied to other DUB targets.

## 1. Introduction

Deubiquitinating enzymes (DUBs) are regulatory enzymes for the ubiquitination pathway. These are grouped into seven distinct sub-families which include the ubiquitin specific proteases (USPs), ubiquitin C-terminal hydrolases (UCHs), Machado Josephin domain proteases (MJDs), ovarian tumor proteases (OTU), Jab1/MPN domain associated metalloisopeptide (JAMM) proteases, and more recently the ZUP1 and MINDY subfamilies [1,2,3].

UCHL3, a member of the UCH subfamily of DUBs, has recently gained traction as a potential cancer target due to its effects on DNA repair pathways and upregulated expression in many cancers. Specifically, UCHL3 has been identified as a deubiquitinase that controls the proteostasis of tyrosyl DNA phosphodiesterase 1 (TDP1), the enzyme responsible for hydrolyzing the covalent bond between type 1 topoisomerases (TOP1) and the 3-prime phosphate of DNA [4]. Depletion of UCHL3 in rhabdomyosarcoma cells markedly reduced the levels of TDP1 and increased sensitization of cells to TOP1 poisons. UCHL3 has additionally been implicated in DNA double stranded break repair pathway by deubiquitinating Ku proteins, which sense broken DNA by binding to chromatin and helping to initiate non-homologous end joining (NHEJ) [5]. Ubiquitination of Ku proteins is important for Ku protein removal from chromatin after NHEJ has completed [6,7]. Other examples include findings that UCHL3 is responsible for deubiquitination of lymphoid-specific helicase (LSH), a chromatin modifier linked to migration, invasion, and tumor formation in non-small cell lung cancer (NSCLC) [8]. Furthermore, overexpression of UCHL3 has also been observed in breast cancer and is well correlated with poor survival rates [9]. Additionally, UCHL3 deubiquitinates and, therefore, stabilizes forkhead box M1 (FOXM1), a key transcription factor and regulator of cell cycle progression in pancreatic cancer leading to cancer progression [10]. Finally, UCHL3 overexpression has been shown to promote ovarian cancer by stabilizing TRAF2 to activate the NF- κB inflammation signaling pathway, leading to poor prognosis for patients [11,12].

While UCHL3 is growing in potential importance relating to its identification as a cancer target, there have been only two small molecule inhibitors reported in the literature. The first reported UCHL3 inhibitor 4,5,6,7-Tetrachloro-1*H*-Indene-1,3 (2*H*)-dione, or TCID, was identified in a high throughput screen to inhibit UCHL3 [13]. However, this was only demonstrated in vitro and the molecule has not been fully validated to inhibit UCHL3 in cells. More recently, the Akt inhibitor perifosine [14] has been suggested to inhibit UCHL3 in breast cancer cell lines [12]. However, this inhibitory effect was demonstrated by deubiquitination of the protein RAD51 in cells by ubiquitin (Ub)-immunoblot but without showing on-target engagement of perifosine with UCHL3 [12]. Furthermore, the molecule does not inhibit UCHL3 in vitro in the standard Ub-AMC enzymatic assays and is known to inhibit other cellular targets, such as protein kinase B [15], limiting its utility as a chemical probe. Based on the lack of UCHL3 chemical probes with validated on-target activity in cells there is a clear need for an alternative strategy to elucidate UCHL3 activity in cancer.

One such alternative strategy is the development of selective Ub-variants (UbVs) for DUBs of interest. Previous work has utilized phage display libraries [16,17,18] or computational [19] approaches, including a report from our group for the design of UCH selective activity-based probes (ABPs) [20], to accomplish this goal. Moreover, the Ovaa group combined both phage-display and computational approached to generate selective UbV activity-based probes for USP family DUBs [21,22]. Another recently reported method for UbV generation utilized combinatorial peptide libraries of the C-terminal motif from Ub to identify unnatural amino acid modifications to the first three residue positions that would impart DUB selectivity, including a UbV selective for UCHL3 [23]

Building on our previous work for computational development of UCH selective UbV-ABPs, we applied a rational design approach to incorporate mutations to the Ub-UCHL3 protein-protein interaction (PPI) interface that would improve binding affinity and impart selectivity over UCHL1 and other DUBs. Our group utilized biolayer interferometry (BLI) and Ub-rhodamine110 (Ub-Rho) inhibition assays to characterize the UCHL3 binding selectivity of our UbV. We then converted the UbV to a UbV-ABP and characterized UCHL3 reaction selectivity both in vitro and cell lysate. The strategy and results for a UCHL3 selective UbV-ABP is presented herein.

## 2. Materials and Methods

### 2.1. Computational Interaction Analysis and Positional Scanning

The Ub-UCHL3 (PDB: 1XD3 [24]) and Ub-UCHL1 (PDB: 3KW5 [25]) complexed crystal structures were imported into Maestro (Schrödinger, LLC, New York, NY, USA) and preprocessing was completed by generating heteroatom states using Epik [26] for the pH range of 7.4 ± 2.0. Hydrogen bond assignments were optimized using PROPKA at a pH of 7.4. Removal of waters at 3.0 Å beyond heteroatoms and with fewer than three H-bonds to non-waters was completed. Energy minimization was completed using OPLS3e force field to yield the minimized structures. These structures were then submitted to BioLuminate (Schrödinger, LLC, New York, NY, USA) [27,28] using the interactions analysis task. A matrix proposed interactions based on the Ub-complex with each DUB was generated and exported for analysis.

Upon analysis of interactions residues were identified on Ub that were at the PPI interface of Ub-UCHL3 but did not contribute productive interactions but did contribute to interactions on the Ub-UCHL1 interface. Ub residue glutamine 40 (Q40) was identified as a position that had little contact with UCHL3 but maintained polar interactions with UCHL1. A residue mutagenesis scan was carried at Q40, in which natural amino acids were mutated in place of Gln and binding affinity was predicted for each Ub-Q40 mutant binding to both UCHL3 and UCHL1 within BioLuminate. A matrix of predicted changes in binding affinity for each Ub-mutant complex was exported and analyzed to prioritize a Q40 mutant for recombinant expression.

### 2.2. Plasmids and Cloning

All plasmids were ordered and generated from GenScript (Piscataway, NJ, USA). UbV site-directed mutagenesis and validation in the pRSET-A monoubiquitin plasmid ordered through GenScript. Plasmids were transformed and expressed as previously described by our group [20].

### 2.3. Recombinant Expression of UCHL3 and UCHL1 Proteins

The recombinant UCHL3 and UCHL1 proteins were recombinantly expressed and purified according to our previously published protocols [20,29,30] and summarized below. A pET-15b plasmid construct was used for the expression of both hexa-histidine (His_6_)-tagged UCHL1 and His_6_-UCHL3 in bacterial culture. These plasmids were transformed into competent BL21 (DE3) E. coli cells using the procedure previously described. Starter cultures were grown at 37 °C with shaking at 250 RPM overnight. 10 mL of starter culture was inoculated into each liter of autoclaved LB media containing 100 µg/mL ampicillin and grown at 37 °C with shaking at 250 RPM to an OD of 0.4–0.8 before being induced with 300 µL of 1.0 M IPTG. These induced cultures were grown for 18 h at 18 °C with shaking at 250 RPM. Bacterial cell pellets were spun down at 4000× *g* for 20 min and resuspended in lysis buffer (1x PBS containing 400 mM KCl). These resuspended bacterial cells were stored in a −80 °C freezer for lysis on a later date or taken directly to lysis by sonication. Lysed bacterial cells were pelleted by centrifugation at 14,000× *g* to remove cellular debris and the supernatant was loaded onto a Nickel-NTA column equilibrated with 1× PBS. After flow through was collected, the column was subject to a 0–500 mM imidazole step gradient and fractions were collected. Both His_6_-UCHL1 and His_6_-UCHL3 eluted from the column at ~150 mM imidazole as evidenced by SDS-PAGE of fractions collected. Fractions that contained the desired protein were pooled together and dialyzed against 1× PBS containing 400 mM KCl with 1.0 mM DTT. This dialyzed protein samples were concentrated down using Amicon Ultra Centrifugal Filters and purified by size-exclusion chromatography (SEC) on an S200 column using running buffer (50mM Tris, 50 mM NaCl, 1mM DTT, pH 7.6). Fractions that contained the protein of interest were concentrated and placed in −80 °C for future experimental use.

### 2.4. Recombinant Expression of UbV Proteins

Mono-ubiquitin variants were purified from a pRSET-A vector as previously described [20,31] and summarized below. The untagged WT-Ub pRSET-A vector construct was obtained from Dr. Das. This WT-Ub plasmid was sent to GenScript where site-directed mutagenesis was performed and validated. All UbVs were purified as described for the UCH proteins with the following changes. The lysis buffer added to the bacterial cell pellets was 50 mM sodium acetate pH = 4.5. After lysis by sonication, the sample was boiled at 80 °C for 5 min to precipitate out the undesired proteins. After centrifugation at 14,000× *g*, the pH of the supernatant was measured to be ~5 so it was adjusted to 4.5 with glacial acetic acid to further precipitate out undesired proteins. The precipitated proteins were centrifuged down at 4000× *g* for 8 min. The supernatant was loaded onto a countertop SP SepharoseTM Fast Flow (Mono S) column (GE Healthcare, product number 17-0729-10), flow through was collected and the column was subject to a 0–1.0 M NaCl step gradient to elute out the UbVs. The fractions that contained UbVs (determined through SDS-PAGE analysis) were concentrated and further purified by SEC on an S200 column as described above.

### 2.5. Ubiquitin Intein Chitin Binding Domain Expression and Purification for Ub-ABPs

The Ub-ABPs were generated according to our previously published protocols [20,25] and summarized below. Variations of ubiquitin-intein-chitin binding domain (Ub-intein-CBD) proteins were expressed in a pTXB1 vector (containing an Mxe intein/chitin binding domain sequence). The process was performed for both WT-Ub and UbV. The WT-Ub-intein-CBD was provided by Dr. Das and additions/mutations were made to this construct and validated by GenScript. Lysis buffer for these expressions was a 300 mM sodium acetate buffer containing 50 mM mercaptoethanesulfonic acid (MES) at pH 6.0 (herein referred to as equilibration buffer). After lysis by sonication, cell debris was pelleted as described above and the supernatant was run in a column containing chitin resin (New England Biolabs, Catalog number: S6651S). Equilibration of the chitin column consisted of running 3 column volume (CV) of equilibration buffer through the column prior to column loading. Another 4 CV of equilibration buffer was washed through the column after which equilibration buffer containing 50 mM MES sodium salt (MESNa) was added. This was incubated in the column for 18 h at 37 °C after which the desired protein was eluted out using the same buffer. The eluted Ub-MESNa sample was concentrated down to ~1.5 mL and stored at −80 °C until further use.

Ub activity-based probes (Ub-ABPs) were constructed by reacting excess glycine-vinylmethyester (VME) or propargylamine (PRG) with Ub-MESNa overnight in 1.0 M sodium bicarbonate containing 150 mg *N*-hydroxysuccinimide (NHS) in a total volume of 10 mL at pH 8.0 (to mitigate MESNa hydrolysis). This was dialyzed into 50 mM sodium acetate buffer pH 4.5 and run on a Mono S column to separate out the Ub-ABP products reacted species. The fractions that contained ubiquitin species of interest were determined by reaction with UCHL1 for 30 min at 37 °C and a subsequent SDS PAGE analysis.

### 2.6. Binding Affinity Analysis

Ub and UbV binding affinity was measured according to a previously reported protocol [19,20] summarized below. This method utilized Ni-NTA coated biosensors (Molecular Devices, Part Number 18-5101) and initial concentrations of the UCH proteins were determined by A280 on NanoDrop™ (ThermoScientific) after which His_6_-UCHL1 and His_6_-UCHL3 were diluted into BLI buffer (1× PBS containing 0.05% *v*/*v* tween 20 and 0.1% *w*/*v* bovine serum albumin (BSA)) to concentrations of 25 µg/mL and 100 µg/mL, respectively, to achieve the similar loading in BLI assay. UbVs were buffer exchanged into 1× PBS using 0.5 mL Zeba™ spin desalting columns (ThermoScientific, catalog number 89882). The concentration of the UbVs was determined by BCA assay and diluted to top concentrations into BLI buffer and 1:1 serial dilutions were completed. Top concentrations differed in assay set-ups based on expected K_d_ from of UbV to UCH protein. 40 µL of each solution was added to a 384 tilted-bottom well plate (Molecular Devices, Part Number 18-5080). One Ni-NTA biosensor was used for each K_d_ measurement, dipping first into BLI buffer (initial baseline, 60 s), then the His_6_-UCH protein wells (loading step, 300 s), then into BLI buffer alone (baseline step, 60 s) followed by dipping into lowest concentration of UbV (association step, 120 s) then into buffer alone (dissociation step, 100 s). A reference sensor of loaded with protein was dipped into buffer only containing wells to adjust for protein-buffer signals. The association-dissociation was repeated with increasing concentration of UbV. All measurements were taken at 30 °C.

Octet Data Analysis Software (version 9.0.0.15) was used to collect and analyze the raw data for the association and dissociation curves. After subtraction of a reference sensor (loaded sensors dipped into buffer only containing wells), averages of the association responses (in nm response signal from 110 s–115 s) was calculated and plotted as a function of UbV concentration in Prism 9. These data were fit to a non-linear regression one site – specific binding model to determine a *K*_d_. Non-specific binding of the sensor to Ub (unloaded sensor tip dipped into Ub containing wells) was checked with WT-Ub. Negligible non-specific signal was observed at a concentration of 2 µM WT-Ub (not shown).

### 2.7. Inhibition Assays

The inhibition assays were carried out as previously described [20,29,30] and summarized below. UbVs were buffer exchanged into 50 mM Tris-HCl containing 0.5 mM EDTA pH 7.6 using 0.5 mL Zeba spin desalting columns (ThermoScientific, catalog number 89882). The concentrations of each UbV were determined by BCA assay and were diluted to the 5× top assay concentrations in activity assay buffer (50 mM Tris, 0.5 mM EDTA, 0.1% bovine serum albumin, 5 mM DTT at pH 7.6). 5× top assay concentrations differed for each UbV based on expected IC_50_. 1:1 serial dilutions of 5× top assay concentrations for each UbVs were completed in activity assay buffer. His_6_-UCHL1 and His_6_-UCHL3 proteins were diluted into activity assay buffer and 20 µL of 2.5 nM His_6_-UCHL1 and 0.25 nM His_6_-UCHL3 were added to wells of a black 384-well plate (Fisher Scientific, Catalog# 12566624, Fisher Scientific, Hampton, NH, USA) and incubated with 10 µL of a 5× concentrations of UbV for 30 min. The difference in enzyme concentration was due to activity differences in the enzymes and necessary to obtain a readout in the linear range for analysis. 450 nM stock of ubiquitin rhodamine110 (Ub-Rho) [32] was made and 20 µL of this stock was added to the assay wells directly before fluorescent measurements were recorded using a Synergy Neo2 Multi-Mode Reader (Biotek, Winooski, VT, USA) at excitation and emission wavelengths = 485 nm and 535 nm, respectively. Initial slopes were identified and plotted using Prism 9. The control (wells containing only activity assay buffer/no ubiquitin inhibitor) was normalized to 100% enzyme activity and the sample wells were calculated at percent activity compared to the control.

### 2.8. Quantification of UbV-ABP Inactivation Efficiency

This method was carried out according to our previously protocols [20] and summarized below. The *K*_inact_/*K*_I_ is a metric that is relevant for irreversible inhibitors as the efficacy of the covalent bond formation is dependent on the rate of the bond forming reaction as well as the ligand binding to the target. The *K*_inact_/*K*_I_ describes the potency of the first reversible binding event in the inhibition constant (*K*_I_) and the maximum rate of inactivation (*K*_inact_). To obtain this data His-UCHL1 and His-UCHL3 enzymes were diluted to 2.5 nM and 0.25 nM stock solutions, respectively, in 50 mM Tris-HCl (pH = 7.6) buffer containing 0.5 mM EDTA, 5 mM DTT, and 0.1% *w*/*v* BSA. HA-WT-Ub-ABPs and HA-UbV-ABPs underwent 1:1 serial dilutions from a top concentration in the same buffer. The UCH enzyme concentrations were optimized to obtain a dynamic range for progress curves for *K*_obs_ determinations. Ub-Rho (Boston Biochem, catalog number U-555) was diluted to 450 nM in the same buffer to make the Ub-Rho stock. 20 µL of Ub-Rho stock solution was first added to each well in a 384-well plate followed by 10 µL of HA-WT Ub ABP or HA-UbV ABP. To initiate the reaction, 20 µL of each respective enzyme stock solution was added and fluorescence measurements were immediately recorded on a Synergy Neo 2 Multi-Mode Reader (BioTek) at excitation and emission wavelengths of 485 nm and 535 nm, respectively. Progress curve raw data was input into Prism 9 and a baseline correction analysis was completed to obtain all the time = 0 points at the origin for fitting purposes. Each progress curve underwent fitting to Y = V_0_ × (1 − $e^{-\left(K_{\mathrm{obs}}\times t\right)}$)/*K*_obs_. [33] The *K*_obs_ values for each progress curve was graphed against the concentration of HA-WT Ub-ABP or HA-UbV-ABP. The slope of the linear fit was determined to be the *K*_inact_/*K*_I_ (the rate constant describing the UbVs inactivation efficiency (covalent bond formation on the catalytic cysteine) on the UCH enzymes resulting from the potency (*K*_I_) of binding and the maximum potential rate of inactivation (*K*_inact_).

### 2.9. Cellular Target Engagement Assays

DUB engagement assays were performed according to previously published protocols. [20,34] Cell pellets were lysed in 50 mM Tris-HCl buffer (pH 7.4) containing 150 mM NaCl, 5 mM MgCl_2_, 5 mM DTT, 2 mM ATP, 0.5% NP-40, and 10% glycerol (herein referred to as cell lysis buffer) for 30 min on ice. Every 10 min the incubating cells were vortexed for 10 s to ensure homogeneous lysis. Cell lysates were clarified by centrifugation at 13,000× *g* for 10 min and the supernatant was collected. Protein concentrations of clarified cell lysates were determined using Bradford assay and each sample was brought to a concentration of 0.5 mg/mL in cell lysis buffer. Initial hemagglutinin (HA) tagged Ub-Activity-Based Probe (ABP = vinyl methylester or propargylamine) concentrations were determined by A_280_ on a NanoDrop™ (ThermoScientific) system and diluted to 10 µM in cell lysis buffer. Concentration determinations by A_280_ measurements were performed with all HA containing Ub and UbVs because of the higher extinction coefficient provided by the HA sequence (leading to more accurate protein concentrations), relative to the mono-ubiquitins. 1 part of 10 µM HA-Ub-ABP was added to 19 parts of 0.5 mg/mL cell lysate and incubated in a heat block at 37 °C for the times stated. 4× Laemmli buffer was added to the samples to terminate the reaction at each timepoint. 10 µL of each sample was loaded onto a 12% SDS-PAGE gel and run at constant 190V for ~75 min. Gels were transferred to a nitrocellulose membrane and subjected to Western blot procedures. Primary antibodies used were HA-Tag-6e2 (Cell Signaling Technologies, Danvers, MA, USA), C29F4 (Cell Signaling Technologies), Ab18181 (Abcam, Cambridge, UK); UCHL3-D25E6 (Cell Signaling Technologies), Ab126621 (Abcam); Alpha Tubulin-Ab7291 (Abcam) or Ab176560 (Abcam). Fluorescent secondary antibodies (Licor IRDye 680RD Goat anti-Rabbit and Licor IRDye 800CW Goat anti-Mouse) were used. Images were collected on a Licor Odyssey system.

## 3. Results

### 3.1. Characterization of T66K/V70F Double Mutant UbV

#### 3.1.1. Inactivation Efficiency of Double Mutants against UCHL3 and UCHL1

Our previous work had identified the UbV with a V70F (UbV^V70F^) mutation to have similar *K*_d_ values in the 7–8 µM range for UCHL3 and UCHL1 but having significantly more selectivity for UCHL3 with regard to inhibitory potency in the Ub-Rho assay [20]. Additionally, our previous work used structural analysis of Ub-USP crystal structures to identify that incorporation of a T66K mutation into UbVs would effectively eliminate binding to USP family DUBs providing a broader selectivity [20]. However, in that work V70F had not been previously combined with T66K and evaluated for DUB activity. We had demonstrated that the T66K mutation had negligible impact on binding affinity or inhibition of either UCHL1 or UCHL3 in our previous work; therefore, because we had already quantified binding affinity and inhibitory activity of the UbV^V70F^, we chose to evaluate the combined UbV T66K/V70F (UbV^T66K/V70F^) in the activity-based probe context. Both UbV^T66K/V70F^-vinylmethyl ester (VME) and -propargyl (PRG) ABPs were generated and evaluated for inactivation efficiency (*K*_inact_/*K*_I_) against both UCHL3 and UCHL1 in vitro and compared to WT-Ub-ABPs (Table 1). It was observed is that UbV^T66K/V70F^-VME, while showing similar inactivation of UCHL3 compared to the WT-UbV-VME, was more than 12,000-fold more efficient at inactivating UCHL3 compared to UCHL1 (*K*_inact_/*K*_I_ = 1.7 × 10^6^ and 140 M^−1^s^−1^, respectively). In fact, the rate of inactivation for UCHL1 was very low indicating the selectivity of the V70F mutation translated from the in vitro binding measurements to the inactivation assays. Interestingly, the UbV^T66K/V70F^-PRG counterpart was less efficient at inactivating UCHL3 compared to both UbV^T66K/V70F^-VME and WT-Ub-PRG, by nearly two orders of magnitude. However, the incorporation of the PRG also resulted in a decrease of inactivation efficiency of two orders of magnitude against UCHL1, thus, leaving UbV^T66K/V70F^-PRG at approximately 10,700-fold more selective for UCHL3 inactivation over UCHL1.

#### 3.1.2. Characterization of UbV^T66K/V70F^-PRG in HEK293 Cell Lysates

**Figure 1:**
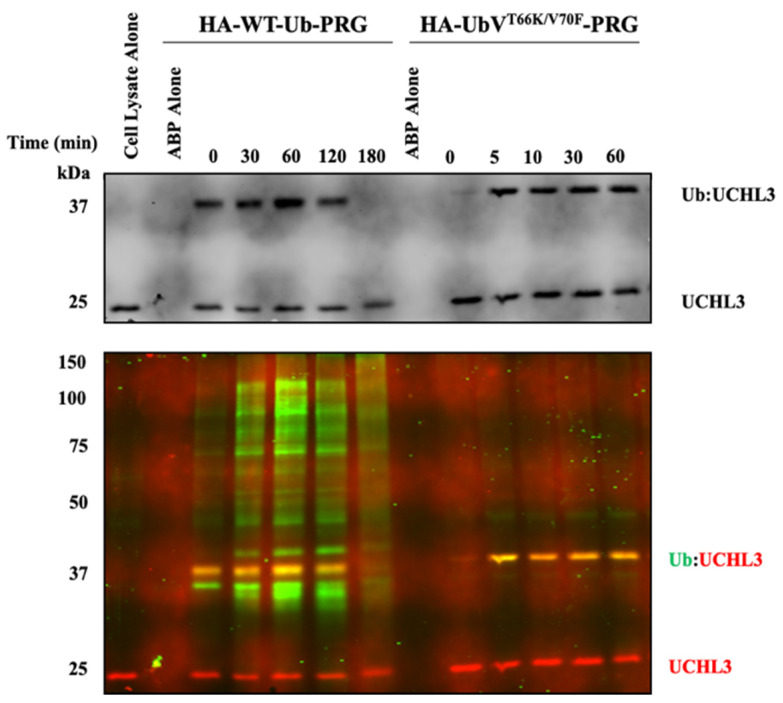
Time-dependent Western blot for Ub-ABPs. HA-WT-Ub-PRG and HA-UbV^T66K/V70F^-PRG, 5 µM, were both dosed into HEK293 lysates (0.5 mg/mL). Samples were tested at the indicated time points. (**Top**) Western blot for UCHL3 indicating the free-UCHL3 and Ub-UCHL3 complex gel shifted. (**Bottom**) Western blot for HA (green fluorescence) and UCHL3 (red fluorescence).

On account that UbV^T66K/V70F^-PRG maintained over 10,000-fold inactivation selectivity for UCHL3 over UCHL1 and because PRG has been reported to be less reactive but more selective for smaller subset of DUBs (including UCHL3) compared to the VME electrophile [35,36] we chose to evaluate this variant for activity in HEK293 cell lysates. For this hemagglutinin (HA)-tagged WT-Ub-PRG and HA-UbV^T66K/V70F^-PRG were dosed at 0.5 µM into HEK293 cell lysates normalized to 0.5 mg/mL total protein concentration, and incubated. Samples were evaluated at different time points by gel mobility shift by immunoblotting for UCHL3 (Figure 1, top) and by co-blotting for HA and UCHL3 (Figure 1, bottom). Both ABPs were shown to interact with UCHL3 as indicated by the presence of the higher molecular weight band correlating to the UCHL3-Ub complex formation over the time course. The HA-UbV^T66K/V70F^-PRG was also shown to be quite selective for UCHL3 compared to the HA-WT-Ub-PRG as there is presence of significant bands of HA-labeled DUBs across a wide range of molecular weights (green fluorescence), whereas only a single band is detected in the HA-UbV^T66K/V70F^-PRG samples (Figure 1, bottom). This band was confirmed to be UCHL3 by co-staining for HA (green) and UCHL3 (red fluorescence) in the same blot. The overlay of the band at 37 kDa is yellow, indicating the HA and UCHL3 signals overlap on that band confirming the UCHL3 selectivity of HA-UbV^T66K/V70F^-PRG over all other DUBs.

### 3.2. Computational Binding Analysis and Positional Scanning

We sought to identify an additional mutation that may be combined with the V70F and T66K to provide improved binding affinity and inhibitory activity for UCHL3 compared to UbV^V70F^ while maintaining the desired selectivity over UCHL1.

To do this a binding analysis of the interactions at the PPI interface of both the Ub-UCHL3 (PDB: 1XD3 [24]) and Ub-UCHL1 (PDB: 3KW5 [25]) crystal structure complexes was carried out using BioLuminate (Schrödinger, LLC, New York, NY, USA) in an effort to identify interactions Ub makes that are different between the two DUBs. This analysis identified three residues on Ub that formed hydrogen bonds with UCHL1 but not with UCHL3, those being Lys6, Glu34 and Gly35. Ub Lys6 forms a hydrogen-bond with Glu35 on UCHL1; however, the corresponding residue on UCHL3 is Asp38. Thus, it would be unlikely to identify a new mutation that would impart improved affinity and selectivity for UCHL3 since both DUBs contain an acidic residue at this location. The same was observed for the interactions formed by Glu34 and Gly35 from Ub to Arg213 on UCHL1 with the corresponding residue on UCHL3 also an arginine.

**Figure 2:**
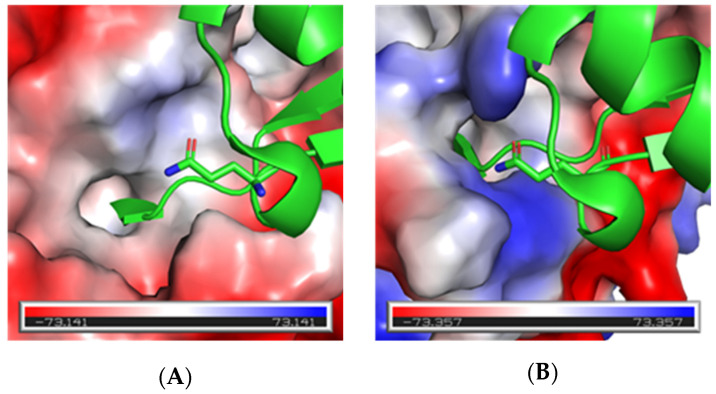
Electrostatic potential surface map representations of Ub (green ribbons) with Q40 (sticks) interaction with UCHL3 (**A**) and UCHL1 (**B**). Electrostatic potential ranges from negative (red) to positive (blue).

We then chose to assess differences with surface interactions of Ub between the two DUBs and this identified Glu40 as a potential candidate for mutagenesis. Residue Gln40 projects toward a pocket of hydrophobic-to-moderate negative electrostatic potential on UCHL3 (Figure 2A). The same analysis showed that Ub Gln40 projects toward the corresponding surface on UCHL1 that is predominantly polar and positively charged (Figure 2B). While Ub Glu40 was not shown to form any productive contacts with UCHL1, the residue side chain was calculated to be 97.5% buried into the complimentary polar pocket. In contrast, the same residue only interacted with the UCHL3 surface with 27% of the accessible side-chain surface area and again formed no productive contacts with the DUB. The lack of polar contacts between Ub Gln40 and UCHL3 led us to hypothesize that mutating this this residue would be tolerated to maintain or possibly improve Ub binding toward UCHL3. Conversely, the residue offered opportunity for mutagenesis that may reduce binding affinity toward UCHL1. The tabular data for the interaction analysis of each PPI is provided as Supplemental information Tables S1 and S2.

With this hypothesis in mind a residue scan was carried out in BioLuminate at Ub Gln40, cycling through all 20 natural amino acid possibilities. This analysis provided predicted ΔAffinity and ΔStability scores for each mutation at Gln40 (provided in Supplemental Materials Table S3). The candidate mutations with the largest difference in ΔAffinity for UCHL3 over UCHL1, as well as the lowest ΔStability value to indicate the mutation providing stable protein were prioritized. This analysis identified hydrophobic mutants at Gln40, particularly Q40L and Q40V mutants, to be predicted to maintain or slightly improve affinity toward UCHL3 while perturbing interaction with UCHL1. The Q40V variant was predicted to be more stable; therefore, this mutant was prioritized to be combined with the V70F/T66K mutations to create a triple-mutant UbV^Q40V/T66K/V70F^ for analysis.

### 3.3. In Vitro Analysis of UbV toward UCHL3 and UCHL1

The triple-mutant UbV^Q40V/T66K/V70F^ was recombinantly expressed and purified for analysis of binding and inhibitory activity against UCHL3 and UCHL1.

#### 3.3.1. Binding Affinity Analysis of Triple-Mutant UbV Using Biolayer Interferometry

**Figure 3:**
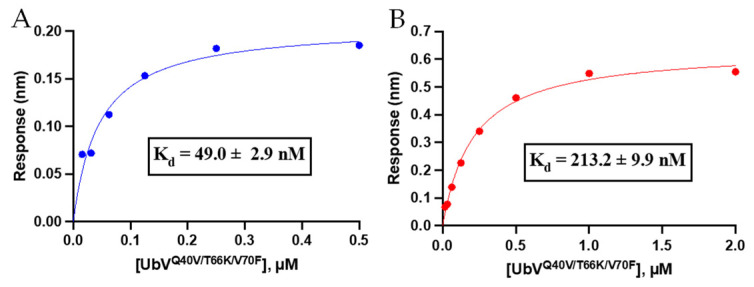
Steady-state binding curves for UbV^Q40V/T66K/V70F^ for determination *K*_d_ using BLI for UCHL3 (**A**, blue) and UCHL1 (**B**, red). Corresponding association/dissociation curves for each replicate are provided in the Supplementary Materials Figure S2.

To assess if the combined effect of the triple mutation in UbV^Q40V/T66K/V70F^ caused improvement for UCHL3 binding and selectivity over our previously published UbV^V70F^ [20], we carried out binding affinity measurements using biolayer interferometry (BLI). It was observed that the triple mutant UbV^Q40V/T66K/V70F^ displayed a steady-state *K*_d_ value of 49.0 ± 2.9 nM for UCHL3 and 213.2 ± 9.9 nM for UCHL1 (Figure 3). Thus, compared to the *K*_d_ values of 7.2 ± 0.5 µM and 8.3 ± 0.1 µM for UbV^V70F^ against UCHL3 and UCHL1 [20], respectively, the triple mutant provided a significant improvement in binding affinity compared to the single V70F mutant for both UCHL3 and UCHL1. All BLI association/dissociation curves are provided in Figure S2. Control with WT-Ub and BLI sensor demonstrated negligible non-specific binding at concentrations up to 2 µM (data not shown). The selectivity also improved from essentially equipotent binding for UbV^V70F^ to both DUBs to >4-fold selectivity for the triple mutant over UCHL3. These data indicate the computational (BioLuminate) approach was feasible to predict a tighter binding UbV for UCHL3; however, the initial hypothesis that introduction of a hydrophobic valine residue at a position that may perturb interaction with a polar region of UCHL1 was not correct. Nonetheless, the overall goal of improved binding affinity and selectivity was achieved.

#### 3.3.2. Biochemical Inhibition of DUB Activity by the Triple-Mutant UbV

**Figure 4:**
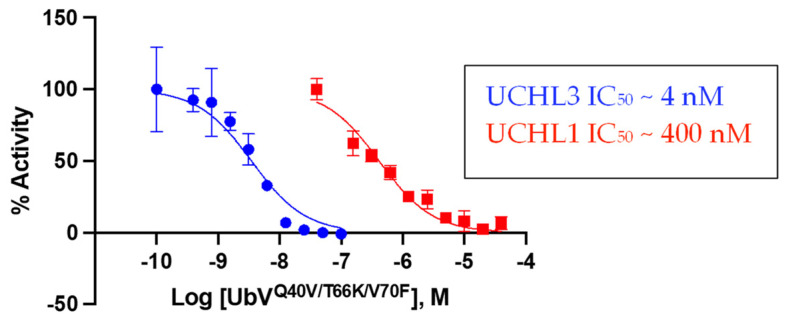
UbV^Q40V/T66K/V70F^ displays selective inhibition of UCHL3. The percent activity of each enzyme normalized to 100% activity for the DMSO control as a function of UbV concentration are plotted for UCHL3 (blue) and UCHL1 (red).

As an orthogonal approach to assess potency and selectivity, UbV^Q40V/T66K/V70F^ was evaluated for inhibitory activity against both UCHL3 and UCHL1 in the Ub-Rho hydrolysis assay [32]. For this particular assay there was a wider selectivity window observed for the variant between the two DUBs. UbV^Q40V/T66K/V70F^ displayed an IC_50_ value of ~4 nM against UCHL3 but nearly 100-fold less potent against UCHL1 with an IC_50_ value of ~400 nM (Figure 4). This data corroborates the BLI data in terms of being selective toward UCHL3. The IC_50_ value against UCHL3 was more potent than the binding affinity value suggesting that UCHL3 catalytic activity in cleaving Ub-Rho is more sensitive to the binding of the triple-mutant UbV. Conversely, the IC_50_ value against UCHL1 was approximately two-fold greater than the *K*_d_ value, indicating that UCHL1 catalytic activity is slightly less sensitive toward the binding of the UbV. In comparison, WT-Ub was more selective for UCHL1 with an IC_50_ value of ~400 nM compared to 820 nM for UCHL3 [20].

#### 3.3.3. Inactivation Efficiency of UbV Triple-mutant Activity-Based Probes

For utility of UbV^Q40V/T66K/V70F^ as an ABP the inactivation efficiency of the UbV containing an electrophilic warhead must be evaluated. We previously observed that the in vitro binding data of UCHL1-selective UbVs did not translate to the same selectivity for UCHL1 upon conversion to UbV-ABPs [20], however, the disconnect was hypothesized to be influenced by the relative reactivity of the catalytic cysteines of UCHL1 and UCHL3. UCHL3 being intrinsically a more active enzyme because the catalytic triad is fully aligned in the apo-state [24], whereas in UCHL1 the triad is misaligned and requires binding of Ub in order to activate the DUB [25]. This is illustrated in the *k*_cat_/*K*_M_ values for the two DUBs with *k*_cat_ = 4.7 s^−1^ for UCHL3 [32] compared to 0.17 s^−1^ for UCHL1 [37].

We observed that the triple mutant UbV^Q40V/T66K/V70F^-VME was similar in reactivity toward UCHL3 compared to WT-Ub-VME (Table 2), an observation that was also noted for the double mutant UbV^T66K/V70F^-VME (Table 1). However, the triple mutant paired with the PRG electrophile was slightly more reactive against UCHL3 with a *K*_inact_/*K*_I_ = 1.8 ± 0.2 × 10^5^ M^−1^s^−1^ compared to double mutant described above (*K*_inact_/*K*_I_ = 4.1 ± 0.4 × 10^4^ M^−1^s^−1^). When evaluated against UCHL1 the triple mutant UbV behaved similarly compared to the double mutant UbV and, in the case of the PRG electrophile, it was slow to react (*K*_inact_/*K*_I_ = 9.1 ± 1.6 M^−1^s^−1^). This resulted in UCHL3:UCHL1 selectivity for UbV^Q40V/T66K/V70F^-PRG of approximately 20,000-fold, almost doubling the selectivity ratio described above for UbV^T66K/V70F^-PRG (10,700-fold). Therefore, the inclusion of the third mutation at Q40, combined with the previous two mutations, improved the potency and selectivity both the UbV and the UbV-PRG for UCHL3 over UCHL1.

### 3.4. Evaluation of UbV^Q40V/T66K/V70F^-PRG in MDA-MB-231 Cell Lysates

**Figure 5:**
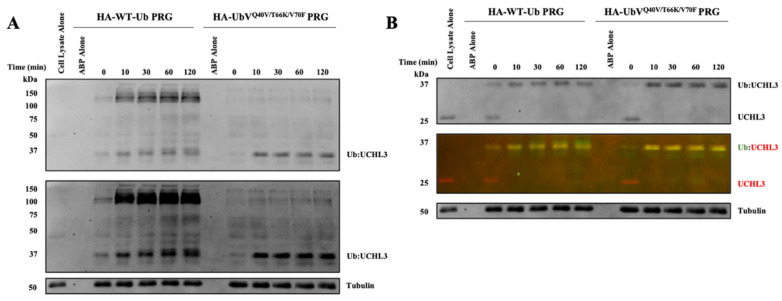
Time-dependent Western blot for Ub-ABPs. HA-WT-Ub-PRG and HA-UbV^Q40V/T66K/V70F^-PRG, 5 µM, were both dosed into MDA-MB-231 cell lysates (total protein concentration of 0.5 mg/mL). Samples were tested at the indicated time points. (**A**) Anti-HA Western blot indicates several non-specific covalent adducts formed by HA-WT-Ub-PRG while the HA-UbV^Q40V/T66K/V70F^-PRG displayed significantly reduced non-specific binding. The blots were imaged with both a normal exposure (top) and dark exposure (bottom) to identify lower concentration or faint bands. (**B**) Anti-UCHL3 and anti-HA colocalization blot that displays the gel-shift of the UCHL3-Ub adducts over time.

Finally, we sought to evaluate the selectivity of the newly developed triple-mutant UbV-PRG for on-target engagement and broader DUB selectivity, this time in a relevant MDA-MB-231 breast cancer cell line. The use of a second cell line, that presumably exhibits different expression patterns of DUBs compared to HEK293 cells, will provide a comparator to assess broad DUB selectivity. DUB inhibition was tracked over the course of 120 min and it was observed that both the HA-WT-Ub-PRG and HA-UbV^Q40V/T66K/V70F^-PRG fully reacted with UCHL3 within the first 10 min of incubation (Figure 5B). However, there was a stark difference in the broad DUB selectivity observed between the two activity-based probes. The WT-Ub-PRG labels several DUBs across a broad range of molecular weights, while the HA-UbV^Q40V/T66K/V70F^-PRG labels apparently a single band that corresponds to the molecular weight of the UCHL3-UbV adduct in the normal exposure blot (Figure 5A, top). A few more bands could be detected in the dark exposure blot (Figure 5A, bottom) but even then, they are significantly lighter than the main band for UCHL3.

## 4. Discussion

Given the lack of quality methods to probe the DUB UCHL3, our group set out to pursue an alternative approach to UCHL3 inhibitor development. Building on previously reported studies to develop UbV for other DUB targets using both experimental mutant screening and computational methods, we employed a computational approach using two different software platforms to arrive at a UCHL3 selective UbV.

First, we started with the UCHL3 UbV^V70F^ that we previously disclosed and demonstrated in vitro selectivity for UCHL3 over the closely related ortholog UCHL1 for inhibition of each DUB in the Ub-Rho assay [20]. We then incorporated the T66K mutation that we found abrogates binding of the USP family DUBs to provide the UbV^T66K/V70F^-ABPs with both PRG and VME as the electrophilic warheads on the C-terminus. The UbV^T66K/V70F^-ABPs demonstrated orders of magnitude selectivity for UCHL3 over UCHL1 in the covalent inhibition kinetics. This may be attributed to an observation previously described by our group [20], in that the catalytic cysteine from UCHL3 is intrinsically more reactive than the UCHL1 counterpart. This is due to the orientation of the catalytic triads from each DUB. UCHL1, in the apo state, has a misaligned catalytic triad that requires the binding of Ub to reorient the triad into a productive confirmation to activate the catalytic Cys [25]. These same dynamics are not necessary for UCHL3 cysteine reactivity as the catalytic triad is already aligned in a productive conformation in the free enzyme [24]. Thus, as evident from comparing the WT-Ub-ABPs, the covalent adducts form at a faster rate with UCHL3 compared to UCHL1. An added level of selectivity was introduced by the choice of the electrophile. The VME electrophile has been shown to be more reactive and rather promiscuous for various DUBs, which could result in off-target non-specific Cys labeling [35]. However, the PRG electrophile was found to be less reactive to non-specific Cys and more selective for a subset of DUBs, including UCHL3 [36]. This same result is observed in the kinetic inhibition data; even though the PRG containing UbVs were less reactive than the VME and WT-Ub counterparts, the PRG containing ABPs were less reactive toward UCHL1 by at least 4 orders of magnitude. Based on this result we moved forward to evaluate HA-UbV^T66K/V70F^-PRG in HEK293 cell lysates and, indeed, the UbV demonstrated superb selectivity for UCHL3 over other DUBs expressed in the HEK cells.

Additionally, we combined a newly predicted Q40V mutation with the prior double mutant to create the triple mutant UbV^Q40V/T66K/V70F^. This data provided mixed results as the triple mutant UbV did indeed improve affinity and inhibition potency against UCHL3, but it also did so against UCHL1. Thus, our hypothesis that we may be able to improve binding affinity and selectivity over the double mutant was only partially correct. However, this may be attributed to the fact that we did not account for Ub already containing the T66K/V70F mutations during the BioLuminate analysis. Perhaps, had we accounted for those existing mutations into the UbV that was utilized for the computational positional scanning we would have identified different mutant to prioritize. Nonetheless, UbV^Q40V/T66K/V70F^ was still more selective for UCHL3 over UCHL1 than the original UbV^V70F^, and we anticipated gaining an extra boost in selectivity when the ABPs were created due to the aforementioned difference in catalytic reactivities between the DUBs, so we took this mutant forward to the ABP stage.

We were delighted to observe that in the case of the PRG ABPs the triple mutant UbV^Q40V/T66K/V70F^-PRG exhibited increased selectivity for UCHL3 over UCHL1 compared to the double mutant counterpart for inactivation efficiency. This selectivity also was demonstrated in the lysate assays, where the ABP proved to be selective for UCHL3 over other DUBs in the MDA-MB-231 breast cancer cells. Unfortunately, a direct comparison cannot be made for selectivity between the double- and triple-mutant UbVs as each were tested in different cell lines; however, we sought demonstrate the on-target engagement and broad selectivity in a cancer relevant cell line. Additionally, the use of a second cell line also allows coverage of an additional cohort of potential DUBs that may be expressed in the MDA-MB-231 cells that would otherwise be absent in HEK293 cells. It should be noted that the claim of UCHL3 selectivity over other DUBs is only relevant to those expressed in these particular cell lines. Further evaluation, including blotting for all DUBs available to assess full target engagement would need to be performed to accurately quantify the level of UCHL3 selectivity. Regardless, the UbV^Q40V/T66K/V70F^-PRG ABP demonstrated significant UCHL3 selectivity in these lysates.

While the feasibility of our approach appears to have been validated, it is not lost on us that the intrinsic reactivity of UCHL3 over UCHL1 is a large contributing factor for the observed ABP selectivity. However, the triple-mutant UbV widened the window of selectivity between the two DUBs when compared to the WT-Ub-PRG. Moreover, in our previous work we also demonstrated that the reduced catalytic activity of UCHL1 indeed worked against our goal of designing a UCHL1 selective UbV-ABP. Thus, in future design of such UbVs, if the goal is to design a selective UbV-ABP, one must first consider the reactivity of the DUB of interest in relation to the closest undesired off-target DUBs. This can be done relatively quickly by measuring the *K*_inact_/*K*_i_ for WT-Ub-ABPs against the DUBs of interest to establish a baseline understanding of how the DUB reactivities compare and take this into account for the subsequent design.

The next phase of such selective UbV-ABPs will be in finding utility for such tools. Thus, the labs that generated the highly potent and selective UbVs could incorporate them into cells via transfection of a plasmid for the mutant UbV sequence and drive expression of said UbV using inducible promoters. This strategy works well if the goal is to inhibit a DUB of interest with the UbV and probe the biological effects. However, in the case of UbV-ABPs one cannot use this strategy as the cell would not be able to append the necessary electrophile to the C-terminus. Therefore, for selective UbV-ABPs to find more use in DUB research strategies to make the ABPs cell permeable must be employed. However, the ability to deliver Ub-based probes to the cellular cytosol has previously been demonstrated using cell-penetrating peptides [38,39,40], which and as a result this may give rise to broader use of UbV-ABPs.

## 5. Conclusions

The work presented herein demonstrates the feasibility of a computational approach for prediction of mutations to Ub that can provide improved binding affinity and selectivity toward DUBs of interest. In this case, the approach was demonstrated on UCHL3 leading to UbV^Q40V/T66K/V70F^-PRG, which exhibited superior selectivity for UCHL3 over UCHL1 in biochemical assays and over other DUBs in cellular lysate-based assays. The results indicate that in the design of DUB selective UbVs the intrinsic reactivity of the DUB of interest should be a primary consideration and that the choice of electrophile can also provide enhanced selectivity. These results provide proof-of-concept studies for design of selective UbV-ABPs against a DUB of interest and discusses the challenges that must be overcome for UbV-ABPs to appeal to the broader scientific community.

## Figures and Tables

**Table 1:** Inactivation efficiency for WT- and double mutant UbV-ABPs versus UCHL3 and UCHL1.

DUB	Electrophile	Ubiquitin	*K*_inact_/*K*_I_ (M^−1^s^−1^) ^a,b^
UCHL3	VME	WT-Ub	1.6 ± 0.07 × 10^6^
		UbV^T66K/V70F^	1.7 ± 0.4 × 10^6^
	PRG	WT-Ub	4.8 ± 0.2 × 10^6^
		UbV^T66K/V70F^	4.1 ± 0.4 × 10^4^
UCHL1	VME	WT-Ub	6.7 ± 0.3 × 10^3^
		UbV^T66K/V70F^	1.4 ± 0.04 × 10^2^
	PRG	WT-Ub	1.3 ± 0.2 × 10^2^
		UbV^T66K/V70F^	3.8 ± 4 × 10^0^

^a^*K*_inact_/*K*_I_ data were extracted from linear regression slopes of graphs of [Ub-ABP] vs. *K*_obs_. The *K*_obs_ values were extracted from a Michaelis-Menten-like fit of progress curve obtained upon incubation of the DUBs with varying concentrations of Ub-ABPs ± standard errors from the [Ub-ABP] vs. *K*_obs_ linear fits (*n* = 3). The progress curves and corresponding linear fits are provided in Supplementary Materials Figure S1. ^b^ WT-Ub progress curves and values previously reported [20].

**Table 2:** Inactivation efficiency for WT- and triple-mutant UbV-ABPs versus UCHL3 and UCHL1.

DUB	Electrophile	Ubiquitin	*K*_inact_/*K*_I_ (M^−1^s^−1^) ^a,b^
UCHL3	VME	WT-Ub	1.6 ± 0.07 × 10^6^
		UbV^Q40V/T66K/V70F^	2.2 ± 0.8 × 10^6^
	PRG	WT-Ub	4.8 ± 0.2 × 10^6^
		UbV^Q40V/T66K/V70F^	1.8 ± 0.2 × 10^5^
UCHL1	VME	WT-Ub	6.7 ± 0.3 × 10^3^
		UbV^Q40V/T66K/V70F^	5.4 ± 0.3 × 10^2^
	PRG	WT-Ub	1.3 ± 0.2 × 10^2^
		UbV^Q40V/T66K/V70F^	9.1 ± 1.6 × 10^0^

^a^*K*_inact_/*K*_I_ data were extracted from linear regression slopes of graphs of [Ub-ABP] vs. *K*_obs_. The *K*_obs_ values were extracted from a Michaelis-Menten-like fit of progress curve obtained after incubating each DUB with varying concentrations of Ub-ABPs ± standard errors from the [Ub-ABP] vs. *K*_obs_ linear fits (*n* = 3). Progress curves and linear fits are provided in Supplementary Materials Figure S3. ^b^ WT-Ub progress curves and values previously reported [20].

## Data Availability

Data is contained within the article or Supplementary Materials.

## Supplementary Materials

The following supporting information can be downloaded at: https://www.mdpi.com/article/10.3390/biom12010062/s1. Figure S1: Progress curves for UbV^T66K/V70F^-ABPs and linear fits, Table S1: BioLuminate PPI interaction analysis for Ub-UCHL3, Table S2: BioLuminate PPI interaction analysis for Ub-UCHL1, Table S3: BioLuminate residue scanning analysis at Gln40 for both Ub-UCHL3 and Ub-UCHL1, Figure S2: BLI association and dissociation curves for UbV^Q40V/T66K/V70F^, Figure S3: Progress curves for UbV^Q40V/T66K/V70F^-ABPs and linear fits.
