# Supplementary material for: Rational Development and Characterization of a Ubiquitin Variant with Selectivity for Ubiquitin C-Terminal Hydrolase L3
Source: Biomolecules. 2022 Jan 1;12(1):62. doi: 10.3390/biom12010062 (PMC8773573; doi:10.3390/biom12010062)
Supplement: Supplementary file 1 [file biomolecules-12-00062-s001.zip › biomolecules-1491685-supplementary.pdf]

Supplementary Information

Rational Development and Characterization of a Ubiquitin Variant with Selectivity for Ubiquitin C-Terminal Hydrolase L3

Chad S. Hewitt [1], Chittaranjan Das [2] and Daniel P. Flaherty [1,3,4,*]

[1] Department of Medicinal Chemistry and Molecular Pharmacology, College of Pharmacy, 575 Stadium Mall Dr. West Lafayette, IN 47907; hewitt8@purdue.edu (C.S.H.), dflaher@purdue.edu (D.P.F.)

[2] Department of Chemistry, College of Science, 560 Oval Dr. West Lafayette, IN 47907; cdas@purdue.edu (C.D.)

[3] Purdue Center for Cancer Research, Hanson Life Sciences Research Building, 201 University St. West Lafayette, IN 47907

[4] Purdue Institute for Drug Discovery, 720 Clinic Ln. West Lafayette, IN 47907

* Correspondence: dflaher@purdue.edu

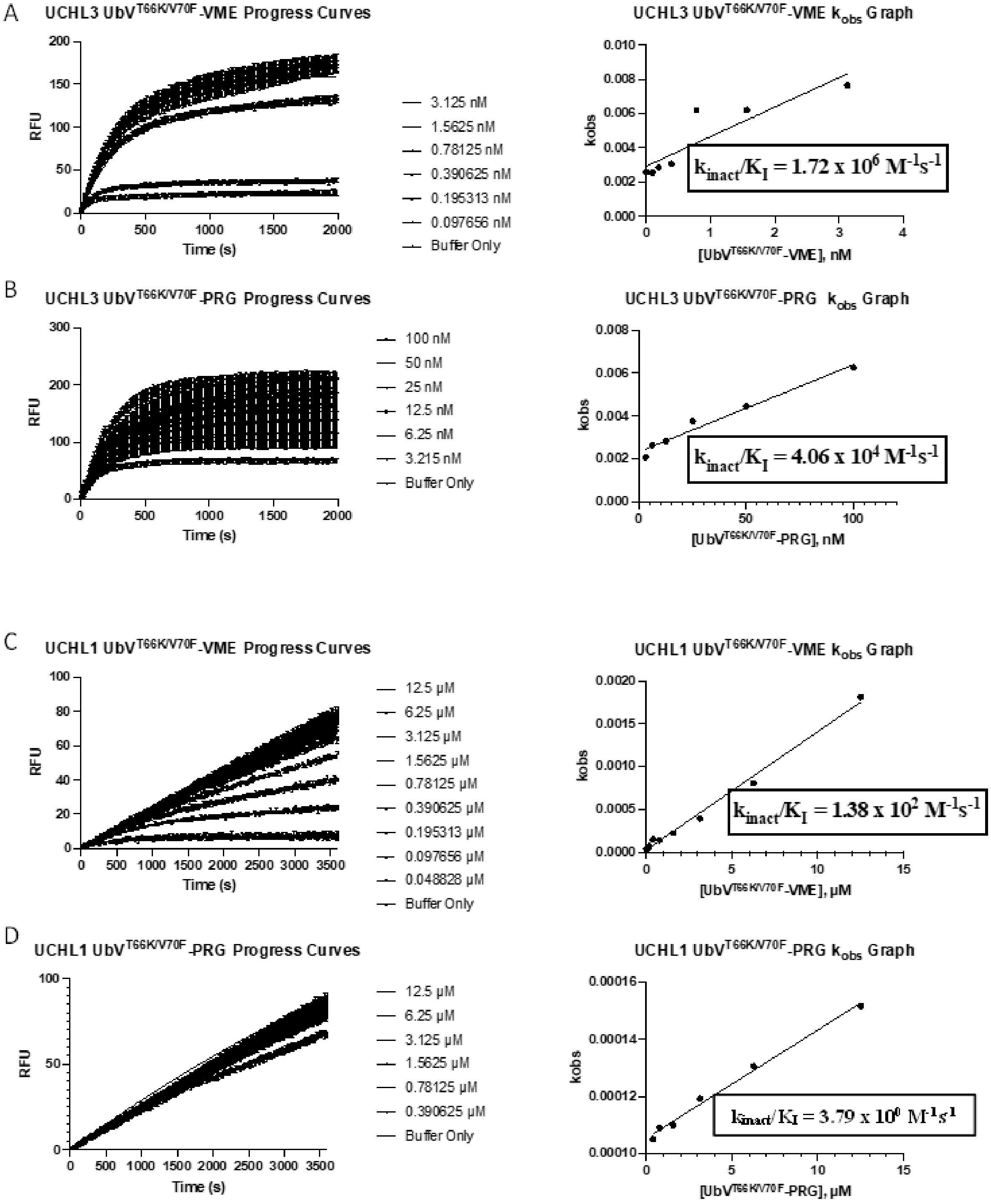

Figure S1. Progress Curves for UbV$^{T66K/V70F}$-ABPs and linear fits k_{obs} vs [UbV-ABP] for against UCHL3 and UCHL1. **A**) UCHL3 with HA-UbV$^{T66K/V70F}$ -VME, **B**) UCHL3 with HA-UbV$^{T66K/V70F}$ -PRG, **C**) UCHL1 with HA-UbV$^{T66K/V70F}$ -VME, **D**) UCHL1 with HA-UbV$^{T66K/V70F}$ -PRG

Table S1. BioLuminate PPI interaction analysis for Ub-UCHL3.

Ub Residue	Closest UCHL3 residue	Distance	Specific Interactions	# HB	# Salt Bridges	# Pi Stacking	# Disulfides	# vdW Clash	Surface Complementarity	Buried SASA
B:6:Lys				0	0	0	0	0	0.2	23.60%
B:7:Thr	A:220:Leu	3.7 A		0	0	0	0	0	0.62	93.30%
B:8:Leu	A:224:Ala<br>A:34:Val<br>A:37:Met<br>A:223:Asn<br>A:36:Gly<br>A:213:Met<br>A:222:Phe	3.4 A<br>3.4 A<br>3.5 A<br>3.7 A<br>3.8 A<br>3.9 A<br>4.0 A		0	0	0	0	0	0.78	99.00%
B:9:Thr	A:220:Leu<br>A:222:Phe<br>A:213:Met	3.4 A<br>3.5 A<br>3.9 A		0	0	0	0	0	0.85	75.20%
B:10:Gly				0	0	0	0	0	0	2.10%
B:11:Lys	A:220:Leu	3.7 A		0	0	0	0	0	0.89	14.70%
B:13:Ile				0	0	0	0	0	0.6	0.00%
B:18:Glu			1x salt bridge to A:108:Lys	0	1	0	0	0	0	0.00%
B:27:Lys				0	0	0	0	0	0	41.10%
B:34:Glu	A:220:Leu	3.2 A		0	0	0	0	0	0.81	21.90%
B:36:Ile				0	0	0	0	0	0.4	53.10%
B:38:Pro				0	0	0	0	0	0	0.10%
B:39:Asp	A:156:Gln<br>A:157:Thr	2.9 A<br>3.3 A	1x hb to A:156:Gln	1	0	0	0	0	0.83	42.90%
B:40:Gln	A:157:Thr	3.8 A		0	0	0	0	0	0.9	27.60%
B:41:Gln	A:156:Gln	2.8 A	1x hb to A:156:Gln	1	0	0	0	0	0.65	100.00%
B:42:Arg	A:156:Gln<br>A:14:Glu	3.1 A<br>3.3 A	1x hb, 1x salt bridge to A:14:Glu<br>1x hb to A:156:Gln	2	1	0	0	0	0.77	58.40%
B:44:Ile	A:35:Tyr	3.6 A		0	0	0	0	0	0.71	99.90%
B:46:Ala	A:44:Met	4.0 A		0	0	0	0	0	0	10.00%
B:47:Gly	A:44:Met<br>A:35:Tyr	3.3 A<br>3.7 A		0	0	0	0	0	0.8	57.00%
B:49:Gln				0	0	0	0	0	0.46	21.70%
B:68:Hie	A:41:Leu<br>A:35:Tyr	3.5 A<br>3.9 A		0	0	0	0	0	0.68	74.50%
B:69:Leu				0	0	0	0	0	0.07	91.70%
B:70:Val	A:223:Asn<br>A:33:Asp<br>A:35:Tyr	3.4 A<br>3.6 A<br>3.7 A		0	0	0	0	0	0.81	96.80%
B:71:Leu	A:223:Asn<br>A:220:Leu	2.9 A<br>3.2 A	1x hb to A:223:Asn	1	0	0	0	0	0.67	98.00%
B:72:Arg	A:33:Asp<br>A:11:Ala<br>A:10:Glu<br>A:12:Asn<br>A:157:Thr<br>A:13:Pro<br>A:156:Gln<br>A:223:Asn	2.8 A<br>3.1 A<br>3.4 A<br>3.4 A<br>3.6 A<br>3.6 A<br>3.8 A<br>4.0 A	1x hb to A:11:Ala<br>2x hb, 1x salt bridge, 1x clash to A:33:Asp	3	1	0	0	1	0.84	99.70%

B:73:Leu	A:11:Ala<br>A:55:Leu<br>A:221:Arg<br>A:10:Glu<br>A:222:Phe<br>A:168:Leu	2.8 A<br>3.4 A<br>3.5 A<br>3.5 A<br>3.7 A<br>3.9 A	1x hb to<br>A:11:Ala	1	0	0	0	0	0.87	98.10%
B:74:Arg	A:152:Ala<br>A:157:Thr<br>A:8:Pro<br>A:154:Glu<br>A:158:Glu<br>A:9:Leu<br>A:160:Pro<br>A:168:Leu<br>A:10:Glu<br>A:166:Val<br>A:159:Ala<br>A:55:Leu	2.8 A<br>2.9 A<br>2.9 A<br>3.1 A<br>3.2 A<br>3.4 A<br>3.5 A<br>3.6 A<br>3.6 A<br>3.6 A<br>3.8 A<br>3.9 A	1x hb to<br>A:8:Pro<br>1x salt bridge<br>to A:10:Glu<br>1x hb to<br>A:152:Ala<br>1x hb to<br>A:154:Glu<br>2x hb to<br>A:157:Thr	5	1	0	0	0	0.87	91.30%
B:75:Gly	A:9:Leu<br>A:8:Pro<br>A:55:Leu<br>A:170:Phe<br>A:168:Leu<br>A:95:Cys	3.1 A<br>3.3 A<br>3.4 A<br>3.5 A<br>3.7 A<br>3.7 A	1x hb to<br>A:9:Leu	1	0	0	0	0	0.9	100.00%
B:75A:Nma	A:168:Leu<br>A:166:Val<br>A:95:Cys<br>A:93:Asn<br>A:8:Pro	3.0 A<br>3.4 A<br>3.4 A<br>3.5 A<br>3.8 A	1x hb to<br>A:168:Leu	1	0	0	0	0	0.84	95.60%

Table S2. BioLuminate PPI interaction analysis for Ub-UCHL1.

Residue	Closest	Distance	Specific Interactions	# HB	# Salt Bridges	# Pi Stacking	# Disulfides	# vdW Clash	Surface Complementarity	Buried SASA
B:6:Lys	A:35:Glu	3.9 A	1x salt bridge to A:35:Glu	0	1	0	0	0	0.13	34.10%
B:7:Thr	A:212:Val<br>A:32:Leu	3.8 A<br>4.0 A		0	0	0	0	0	0.5	98.50%
B:8:Leu	A:31:Val<br>A:34:Leu<br>A:215:Ser<br>A:33:Gly<br>A:216:Ala<br>A:32:Leu<br>A:214:Phe	3.4 A<br>3.4 A<br>3.5 A<br>3.6 A<br>3.6 A<br>3.7 A<br>3.9 A		0	0	0	0	0	0.84	98.60%
B:9:Thr	A:214:Phe<br>A:205:Thr	3.4 A<br>3.6 A		0	0	0	0	0	0.82	83.50%
B:10:Gly				0	0	0	0	0	0.04	16.10%
B:11:Lys	A:212:Val	3.8 A		0	0	0	0	0	0.72	24.80%
B:13:Ile				0	0	0	0	0	0.77	1.40%
B:34:Glu	A:213:Arg<br>A:212:Val	3.2 A<br>3.4 A	1x hb to A:213:Arg	1	0	0	0	0	0.85	45.40%
B:35:Gly	A:213:Arg	3.1 A	1x hb to A:213:Arg	1	0	0	0	0	0.86	16.60%
B:36:Ile	A:213:Arg<br>A:212:Val	3.5 A<br>3.8 A		0	0	0	0	0	0.65	100.00%
B:37:Pro	A:154:Val<br>A:213:Arg	3.7 A<br>3.9 A		0	0	0	0	0	0.5	79.40%
B:39:Asp	A:153:Arg	3.5 A		0	0	0	0	0	0.73	28.30%
B:40:Gln	A:153:Arg<br>A:155:Asp	3.4 A<br>3.6 A		0	0	0	0	0	0.77	97.50%
B:42:Arg				0	0	0	0	0	0	20.20%
B:44:Ile				0	0	0	0	0	0.75	60.10%
B:47:Gly				0	0	0	0	0	0	9.90%
B:49:Gln				0	0	0	0	0	0	3.10%
B:68:His	A:32:Leu<br>A:38:Ser	3.7 A<br>3.8 A		0	0	0	0	0	0.58	64.20%
B:69:Leu				0	0	0	0	0	0.09	93.10%
B:70:Val	A:32:Leu<br>A:30:Asp	3.5 A<br>3.9 A		0	0	0	0	0	0.76	93.00%
B:71:Leu	A:215:Ser<br>A:212:Val<br>A:8:Ile<br>A:213:Arg<br>A:214:Phe	2.9 A<br>3.5 A<br>3.9 A<br>3.9 A<br>3.9 A		0	0	0	0	0	0.87	96.70%

B:72:Arg	A:30:Asp<br>A:153:Arg<br>A:9:Asn<br>A:8:Ile<br>A:7:Glu<br>A:10:Pro	2.8 A<br>2.9 A<br>3.2 A<br>3.2 A<br>3.4 A<br>3.7 A	1x hb to A:8:Ile<br>1x clash to A:9:Asn<br>2x hb, 1x salt bridge, 1x clash to A:30:Asp<br>1x clash to A:153:Arg	3	1	0	0	3	0.86	97.00%
B:73:Leu	A:8:Ile<br>A:52:Leu<br>A:213:Arg<br>A:7:Glu<br>A:6:Met<br>A:215:Ser	3.0 A<br>3.4 A<br>3.5 A<br>3.5 A<br>3.7 A<br>3.9 A	1x hb, 1x clash to A:8:Ile	1	0	0	0	1	0.82	100.00%
B:74:Arg	A:155:Asp<br>A:151:Gln<br>A:160:Phe<br>A:156:Asp<br>A:6:Met<br>A:153:Arg<br>A:5:Pro	3.0 A<br>3.2 A<br>3.4 A<br>3.5 A<br>3.5 A<br>3.9 A<br>3.9 A	1x hb to A:155:Asp	1	0	0	0	0	0.78	83.50%
B:75:Gly	A:6:Met<br>A:5:Pro<br>A:52:Leu<br>A:162:Phe<br>A:160:Phe<br>A:90:Cys	2.9 A<br>3.6 A<br>3.6 A<br>3.7 A<br>3.8 A<br>3.9 A	2x hb to A:6:Met	2	0	0	0	0	0.8	100.00%
B:75A:Nma	A:160:Phe<br>A:90:Cys<br>A:88:Asn	2.9 A<br>3.4 A<br>3.6 A	1x clash to A:90:Cys<br>1x hb to A:160:Phe	1	0	0	0	1	0.65	91.60%

Table S3. BioLuminate Residue Scanning Analysis at Gln40 for both Ub-UCHL3 and Ub-UCHL1

Gln40(X); X =	$\text{Affinity}_{\text{UCHL3}}$ (kcal/mol)	$\text{Stability}_{\text{UCHL3}}$ (kcal/mol)	$\text{Affinity}_{\text{UCHL1}}$ (kcal/mol)	$\text{Stability}_{\text{UCHL1}}$ (kcal/mol)
**Ala**	1.0	3.6	6.9	0.2
**Arg**	-1.2	-2.1	-6.0	3.7
**Asn**	-0.3	3.1	4.1	3.0
**Asp**	6.8	7.8	10.2	9.3
**Cys**	0.0	5.7	4.4	2.4
**Glu**	4.9	-1.5	5.4	-2.1
**Gly**	1.7	11.5	7.9	8.0
**Hid**	-1.1	-1.5	4.6	11.9
**Hie**	-0.7	3.9	3.4	16.3
**Hip**	-5.5	0.8	8.1	11.5
**Ile**	-1.0	-8.7	1.5	-6.6
**Leu**	-1.1	-2.7	4.7	1.1
**Lys**	-0.9	13.4	7.6	17.6
**Met**	-2.0	-11.3	0.2	-11.1
**Phe**	-2.8	0.8	1.2	-0.1
**Pro**	-0.2	31.1	3.8	32.2
**Ser**	0.4	6.8	5.0	3.0
**Thr**	-0.1	1.4	5.0	-1.6
**Trp**	-4.2	6.8	75.0	140
**Tyr**	-2.3	0.8	11.3	65.5
**Val**	-0.6	-6.0	4.1	-3.6

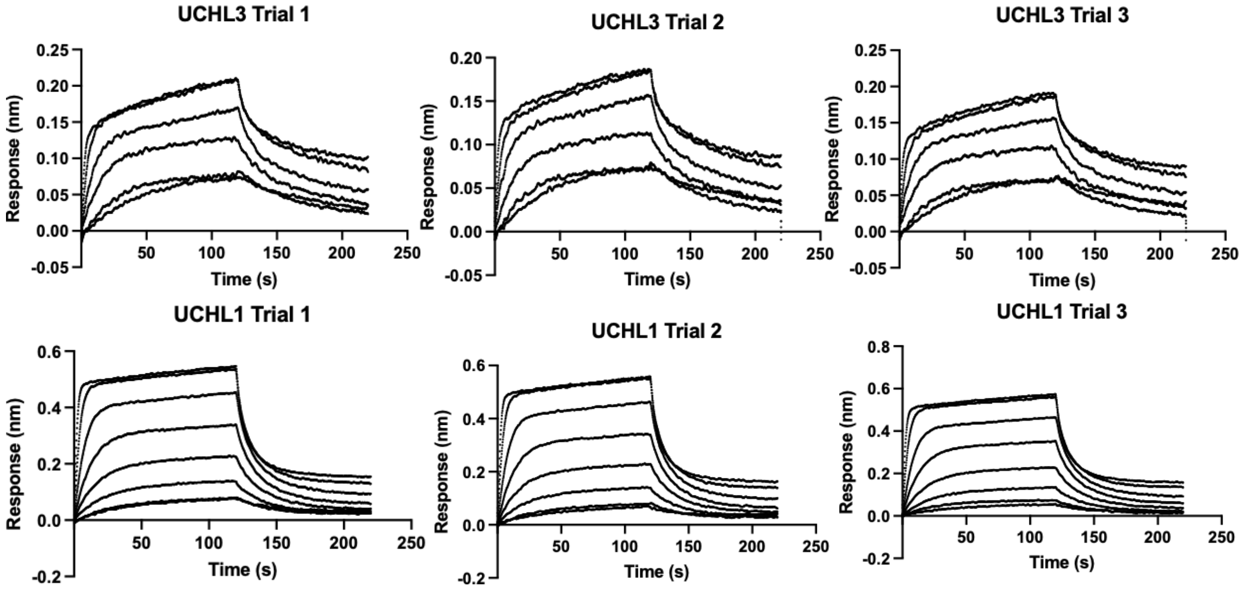

Figure S2. BLI association and dissociation curves for UbV$^{Q40V/T66K/V70F}$ against UCHL3 (**top row**) and UCHL1 (**bottom row**). The maximum response was plotted as a function of concentration of UbV to generate steady-state binding curves in main text.

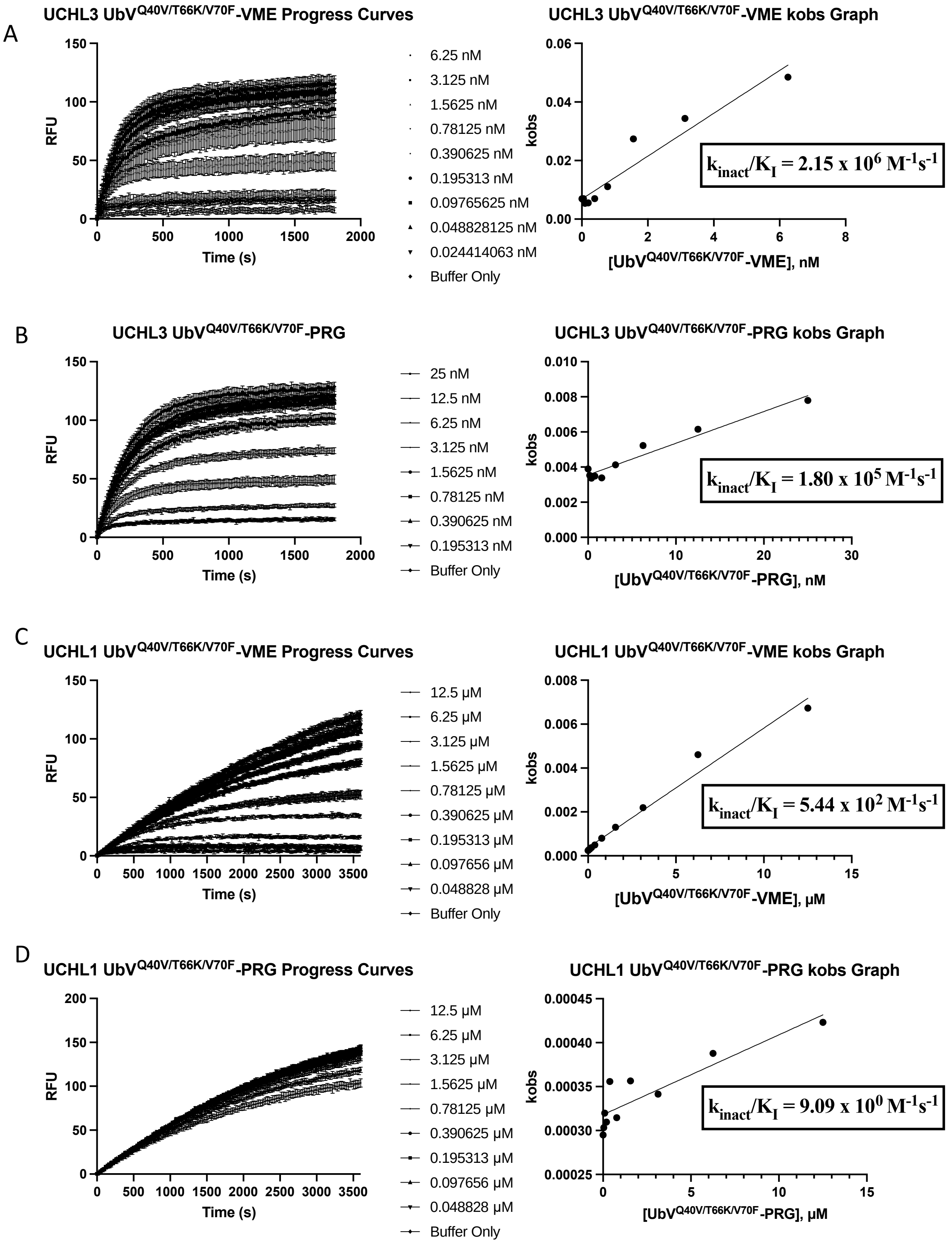

Figure S3. Progress Curves for UbV$^{Q40V/T66K/V70F}$-ABPs and linear fits k_{obs} vs [UbV-ABP] against UCHL3 and UCHL1 with **A**) UCHL3 with HA-UbV$^{Q40V/T66K/V70F}$-VME, **B**) UCHL3 with HA- UbV$^{Q40V/T66K/V70F}$-PRG, **C**) UCHL1 with HA- UbV$^{Q40V/T66K/V70F}$-VME, **D**) UCHL1 with HA- UbV$^{Q40V/T66K/V70F}$-PRG
